# Microhabitat Patchiness Structures Benthic Biodiversity in the Western Antarctic Peninsula

**DOI:** 10.1002/ece3.73392

**Published:** 2026-04-01

**Authors:** Lea Katz, Emily Mitchell, Bruno Danis, Huw Griffiths

**Affiliations:** ^1^ Marine Biology Laboratory Université Libre de Bruxelles Brussels Belgium; ^2^ Deep‐Time Ecology Group, Department of Zoology University of Cambridge Cambridge UK; ^3^ British Antarctic Survey Cambridge UK

**Keywords:** Antarctic Peninsula, benthos, biodiversity, microhabitats, underwater imagery

## Abstract

In the accelerating global biodiversity crisis, it is imperative to document biodiversity patterns along with their underlying drivers and the processes driving species distributions. Our objective was to study how benthic community composition varies across spatial scales, and how fine‐scale patchiness contributes to larger scale biodiversity in the Western Antarctic Peninsula. Using underwater imagery, we quantified benthic community composition at three different scales using a nested sampling design. We used multivariate analyses (CLUSTER, NMDS, ANOSIM, SIMPROF and SIMPER analysis) to detect patterns in benthic community variability. NMDS showed that images were clustered according to their community composition into 31 significant groupings, or microhabitat types, revealing high variability. These microhabitats were not restricted by geographic location, substrate type or macroalgal cover, as initially presumed. Instead, the differences in faunal assemblage seem to be influenced by the dominant macroalgae species and biogenic habitat complexity. We found that these microhabitats emerge as ecologically meaningful units that offer a practical scale at which to detect patterns and early ecological shifts. Changes in the distribution or frequency of these microhabitats may occur before shifts in species composition become detectable at site or station level, which is crucial for ecosystem monitoring.

## Graphical abstract

Shallow benthic communities of the West Antarctic Peninsula cannot be considered uniform habitats but rather mosaics of microhabitats, each characterised by distinct macroalgal communities and associated fauna. Regional biodiversity and patterns are shaped by the number and types of microhabitats. Changes in their distribution may signal ecological change before shifts in species composition become detectable at site or station level.

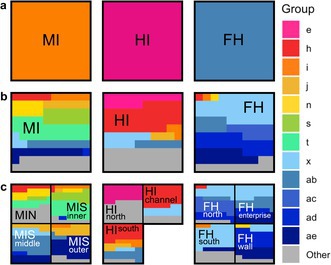

## 1 Introduction

Marine biodiversity is important for maintaining ecological equilibrium of oceanic ecosystems, providing essential services (Goulletquer et al. 2014), but it is currently under significant threat (Duterte 2025). Habitat complexity and heterogeneity are key drivers of biodiversity, enhancing abundance, taxonomic richness and community dissimilarity (Thomsen et al. 2022; Leite Jardim et al. 2025). While much of this complexity arises from physical factors such as substrate type and topography, habitat‐forming organisms like macroalgae and sponges also contribute significantly to structural heterogeneity by creating 3D complexity that supports diverse associated communities (Gutt et al. 2017; Rossi et al. 2017).

Despite the growing evidence that spatial heterogeneity enhances biodiversity, most ecological studies and predictive models are constrained to single spatial or temporal scales (Teng et al. 2020). This limitation risks overlooking fine‐scale variability and multi‐scale processes that may be ecologically meaningful (Zelnik et al. 2024). Large scale analysis may either miss early signals of ecological change or obscure them through spatial averaging, masking the rich patterning of ecological responses (Tielens et al. 2019; Zelnik et al. 2024). These missing signals may be particularly problematic in rapidly changing systems where detecting subtle, early shifts can inform timely management responses.

The Western Antarctic Peninsula (WAP) presents a unique framework to study biodiversity patterns in a system with little to no direct human disturbance, due to strict regulation by the Commision for the Conservation of Antarctic Marine Living Resources (CCAMLR). This protection allows ecological processes to be examined in relative isolation from confounding anthropogenic impacts. The northern part of the WAP is characterised by a shorter sea‐ice season, a higher sea surface temperature than more southerly parts of Antarctica, a maritime climate with higher precipitation, coastal near‐shore meltwater runoff and year‐round light availability (although much reduced during the winter months) compared to the south (Matsuoka et al. 2021).

The WAP is undergoing rapid environmental change (Turner et al. 2014; Chown et al. 2022), particularly in warming and sea‐ice dynamics (Siegert et al. 2019; Eayrs et al. 2021). Along the northern WAP (extending from the South Shetland Islands to around 65°S), winter sea‐ice cover has decreased by more than 3 months, meaning an almost total loss of winter sea‐ice cover. In the southern WAP and Bellingshausen Sea, sea ice retreat is over a month earlier and advance up to 2 months later, leading to significantly longer ice‐free conditions (Stammerjohn et al. 2008, 2012; Matsuoka et al. 2021). Such changes to the sea ice regime are known to affect benthic communities through a combination of increased light availability and increased iceberg mobility and scour, leading to negative consequences for animal‐dominated, sessile, shallow water communities (Smale 2008; Pasotti et al. 2015; Clark et al. 2017; Gutt et al. 2019; Amsler et al. 2023; Griffiths et al. 2024). These changes are predicted to intensify in coming decades, with further consequences for benthic communities along the WAP and into deeper water (Griffiths et al. 2024).

The WAP is recognised as a hotspot for benthic organisms (Gutt et al. 2016), and despite the importance of fine‐scale heterogeneity, benthic habitats of the WAP are poorly documented (Gutt et al. 2019), thus representing a key knowledge gap in understanding spatial biodiversity patterns in polar marine ecosystems. Furthermore, this lack of data poses challenges for conservation efforts which require robust information for the designation of Marine Protected Areas in the framework of CCAMLR and the Antarctic Treaty Consultative Meeting—Committee on Environmental Protection (ATCM‐CEP).

Using analyses of underwater imagery from the shallow coastal waters of the WAP, we investigate how benthic community variability is structured across three different spatial scales, using a nested sampling design and multivariate analyses. We hypothesised that different patterns would be observed at different scales, with regional patterns being determined by large scale drivers such as sea ice cover and small‐scale patterns determined by local conditions such as substrate type and level of macroalgal cover.

## 2 Materials and Methods

### 2.1 Sampling Stations

**FIGURE 1:**
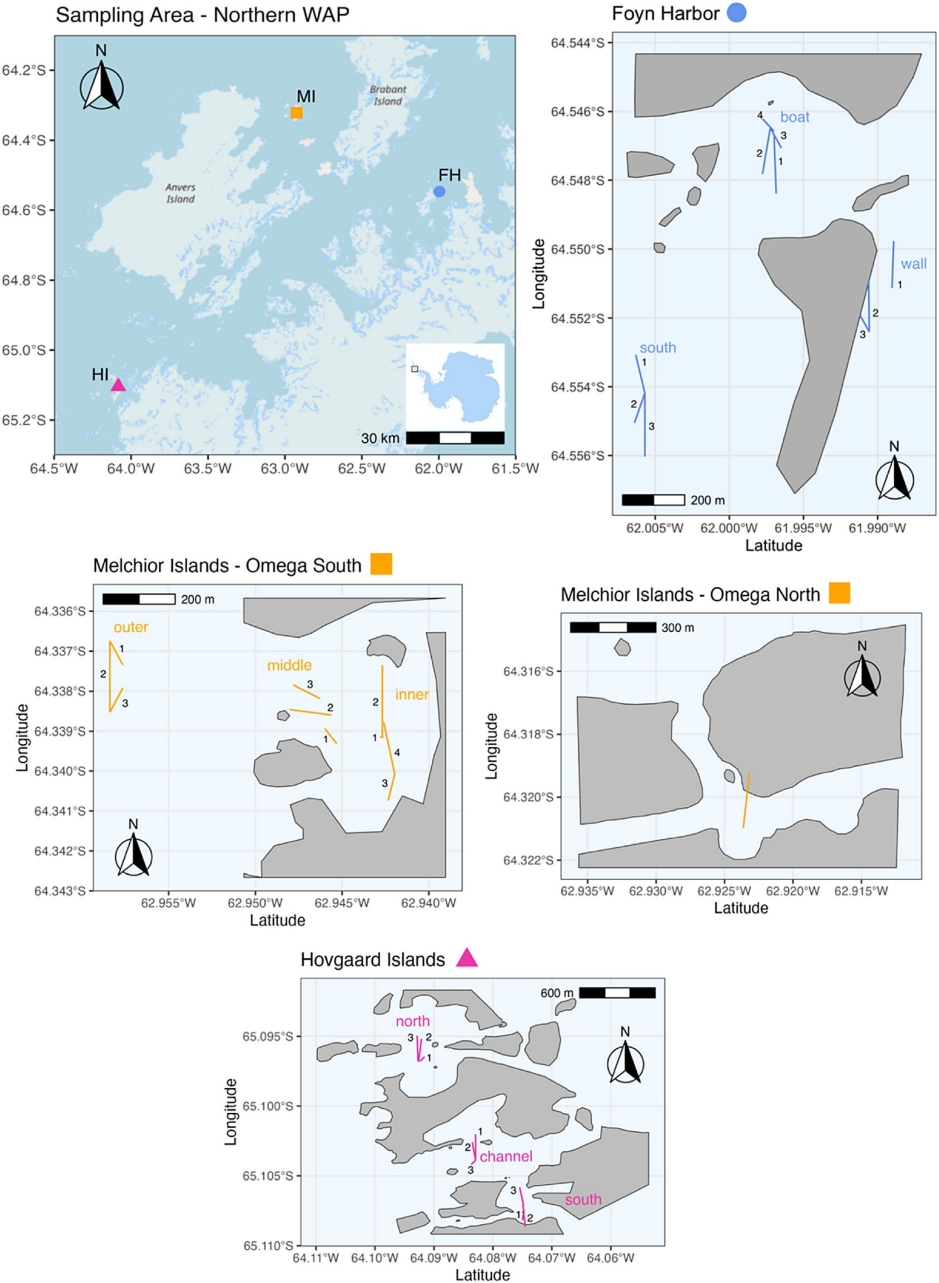
General map of sampling area (Northern Wap) and detailed maps of sampling stations (Melchior Islands Omega North and South, Hovgaard Islands and Foyn Harbour) with locations of sampling sites and transects. FH‐e is not represented on the map but is situated in north‐east from FH‐b (“boat”). A fourth site, near to Enterprise Island and (“FH‐e”), north of Foyn Harbour is beyond the scope of this map and not shown.

All sampling events were undertaken during the second expedition of the TANGO project that took place between February and March 2024 in the Western Antarctic Peninsula (WAP). For this second cruise, the crew focused on the Northern part of the WAP aboard a sailing boat, the RV Australis (Danis et al. 2024). During this cruise, three stations of interest were selected: Melchior Islands, Hovgaard Islands and Foyn Harbour, with 50 to 100 km between each station (Figure 1 and Table 1). The Melchior Islands are a complex of islands lying between Brabant Island and Anvers Island. Sampling took place around the biggest island, Omega Island (Figure 1). Foyn Harbour, an active centre for whaling operations until the 1930s, is an area with a few islands including Enterprise Island and the wreck of the Guvernøren.

**TABLE 1:** Description of ROV stations undertaken during the second expedition of the TANGO project that took place between February and March 2024.

Station	Site	Latitude	Longitude	Depth range (m)
Melchior Islands		64°19.246 S	62°55.375 W	16–41
	Melchior Islands—North Omega (MIN)			16
	Melchior Islands—South Omega Inner Bay (MIS‐I)			21–22
	Melchior Islands—South Omega Middle Bay (MIS‐m)			29–40
	Melchior Islands—South Omega Outside Bay (MIS‐o)			22–41
Hovgaard Islands		65°06.057 S	64°04.992 W	12–34
	Hovgaard Islands—North (HI‐n)			34
	Hovgaard Islands—South (HI‐s)			12–13
	Hovgaard Islands—Channel (HI‐c)			19–20
Foyn Harbour		64°32.798 S	61°59.885 W	10–53
	Foyn Harbour—Boat Anchor (FH‐b)			21–24
	Foyn Harbour—South (FH‐s)			22–37
	Foyn Harbour—Wall (FH‐w)			26–53
	Foyn Harbour—Enterprise Island (FH‐e)			10–24

For our sampling strategy we opted for a nested design to integrate the effect of sampling scale on the survey. The broadest geographic category analysed was the stations: Melchior Islands, Hovgaard Islands and Foyn Harbour (Figure 1 and Table 1). Nested within the stations were the different sampling sites. Each site within a station was separated by a maximum of 1 km (Figure 1 and Table 1) and was usually a pool‐like area. Several transects were surveyed in different directions within each site (Figure 1). Our smallest unit of investigation was the individual photographs taken along each transect.

### 2.2 Image Acquisition

Underwater image sampling was done using a small Remotely Operated Vehicle (BlueROV2, Heavy configuration) equipped with a GoPro HERO10 camera capturing videos of the seafloor. The BlueROV was deployed from either the main vessel or a zodiac and flown in linear transects at a constant altitude (approximately 1 m from the bottom, adjusted for visibility, using the ROV's ping sonar altimeter) and a very slow pace (< 1 m/s) to maintain a high video quality. The total area of each analysed photograph was ~0.8 m^2^. Depending on the topography and deployment vessel, transects were either evenly spaced out in the sampling site, in a zig‐zag pattern, or started from the same point and went in different directions, but never overlapping in both cases (Figure 1). Transect length varied from 25 to 110 m.

### 2.3 Image Analysis

For each video transect, still images were extracted using FFmpeg (Bellard 2023). And a set of evenly spaced‐out images was selected for annotation from each transect, with a minimum of 30 images per site. The selection of the images was done automatically based on time‐separation, but if an image was blurry, the closest non‐blurry image was manually selected.

Image annotation was performed in BIIGLE (Langenkämper et al. 2017). In each image, all visible animals, algae and substrate features were recorded using the CATAMI label tree (Althaus et al. 2015) with supplementary branches specific to Antarctic biota (Katz 2024). Since the identification level varied among taxa (species, genus or family), we use the term “morphotaxa” to refer to the identified biota throughout this study. Given the distance, resolution of images, habitat type and size and colour of some taxa, many taxa will not have been observable or fully accounted for. The example images (Table S2) demonstrate that sub‐centimetre sized organisms, those with camouflage or cryptic habitat choices, or any infauna are unlikely to be well represented, if at all. Whilst other collection methods were used during the second TANGO expedition, only organisms that could be confidently observed from overhead photography were analysed for this study.

For more common morphotaxa, we were able to confidently assign species as some were hand‐picked by SCUBA divers (Danis et al. 2023). However, when labels lacked the precision required for this study, we chose to omit the corresponding images. The resulting abundance dataset comprised 336 annotated photos which included 2410 individual annotations, 91 identified CATAMI categories (of which 13 were substrate features). Individual animals were kept as counts, but the abundance of macroalgae and colonial animals was converted into percentage (%) cover.

To simplify the substrate types, they were classified into four categories: soft (which included CATAMI labels “sand_mud”, “fine_sand” and “coarse_sand”), hard (including CATAMI labels “cobbles”, “gravel”, “pebble”, “consolidated”, “boulder”, “rock”), mix, and obscured (substrate was not visible in more than 70% of the image due to being obscured by organisms). Where possible, substrate type was confirmed by scientific divers. To compare the different sampling scales, abundances were summarised per image, transect, site and station.

### 2.4 Statistical Analysis

Abundance data was transformed to square root to suitably weigh the importance of the very abundant morphotaxa and to increase the effect of rarer morphotaxa. Similarity between images was calculated using the Bray–Curtis similarity coefficient.

Hierarchical clustering on the images was performed in R (version 4.3.1, R Core Team 2023) and a SIMPROF analysis at a 5% significance level was run in PRIMER (version 7, Clarke and Gorley 2015) to determine statistically significant groups. The purpose of the SIMPROF test is to test the hypothesis that within the individual photographs dataset there is no evidence of multivariate structure. It sequentially tests the nodes of the dendrogram until the null hypothesis is rejected, providing evidence of multivariate structure.

Three different Nonmetric MultiDimensional Scaling (NMDS) ordinations were performed in R using the vegan package (Oksanen et al. 2001) at three different spatial scales, changing the sample unit each time (image, transect, and site). For the image‐scale NMDS, two outliers (1 image from HI and 1 image from MI) were removed from the graph for more clarity.

Analysis of similarities (ANOSIM) was performed on the relationships between community composition and location, percentage macroalgal cover and substrate type.

A Similarity Percentage (SIMPER) analysis was run in R between the most common groups from the SIMPROF analysis (***e, h, i, j, n, s, t, x, ab, ac, ad***). These groups represent habitats of small or of limited extent, differing in character from each other or the surrounding habitats. Such habitats are widely known as microhabitats, and henceforth these groupings will be referred to as such in this paper. For each microhabitat, the morphotaxa contributing up to 70% to the overall microhabitat resemblance were calculated, and a short description of the community was written in Table S1, along with example images.

## 3 Results

### 3.1 Morphotaxa and Microhabitat Diversity

At the station level, Melchior Islands (MI) has the highest number of morphotaxa (53) and microhabitats (19), but the individual sites are poorer with between 18 and 37 morphotaxa (Table S2 and Figure S1). This community composition results in MI having the lowest Shannon‐Wiener diversity and evenness scores at both station (1.611 and 0.405) and site level (between 0.625 and 1.693 and between 0.216 and 0.533) (Table S2 and Figure S1a,b). With a lower number of microhabitats (17), Hovgaard Islands (HI) has the lowest total number of morphotaxa (45) but hosts diverse communities at site and station level (Table S2 and Figure S1) and the highest evenness of the three stations (0.611). Foyn Harbour (FH), with 12 microhabitats and 45 morphotaxa, is not the most biodiverse (2.117) or even (0.541) at the station scale but has the highest biodiversity metric for sites (between 1.775 and 2.072, Table S1 and Figure 1). This is due to FH being dominated by the most diverse communities (Table S1 and Figure 1).

### 3.2 Substrate

In general, both the northerly stations, MI and FH, substrate composition was relatively evenly mixed, including all substrate types, soft and hard, and no dominant type (Table 2). One exception to this pattern was the MIS‐i site, where the substrate was either unconsolidated sediment or obscured by macroalgae (37% and 63%, respectively), with no rocky substrate observed. Likewise, the southerly station, HI, was dominated by unconsolidated soft sediments (> 73%), with < 1% hard substrate in the form of dropstones (ice‐rafted rocks or stones). The FH‐w and MIS‐o sites were dominated by hard substrate, with < 5% soft substrate visible.

**TABLE 2:** Summarised dominant substrate types and % macroalgal cover for the analysed seafloor images taken during the second expedition of the TANGO project.

Station	Site	Number of images analysed	Dominant substrate (% of images)				Mean % macroalgal cover
			Hard	Soft	Mix	Obscured	
Melchior Islands		154	24.7	27.9	2.6	44.8	67.3
	MIN	44	29.5	29.5	4.5	36.4	69.5
	MIS‐I	65	0	36.9	0	63.1	74.5
	MIS‐m	24	25	20.8	8.3	45.8	71.8
	MIS‐o	21	90.5	4.8	0	4.8	35.4
Hovgaard Islands		79	0	73.4	7.6	19	35.1
	HI‐n	28	0	67.9	10.7	21.4	45.5
	HI‐s	21	1	80	9.5	9.5	23.1
	HI‐c	30	0	73.3	3.3	23.3	33.8
Foyn Harbour		103	33	21.4	30.1	14.6	50.7
	FH‐b	29	31	20.7	24.1	24.1	59.7
	FH‐s	26	11.5	30.8	57.7	0	29.2
	FH‐w	25	84	4	0	12	50.4
	FH‐e	23	8.7	30.4	39.1	21.7	63.7

### 3.3 Macroalgae

MI exhibited the highest overall macroalgal cover among the stations (ranging from 40% to 65% across sites), closely followed by FH, which also showed consistently high values across sites (35%–74% across sites). In contrast, HI had substantially lower total macroalgal cover (23%–45% across sites). Sites at the northerly MI station recorded macroalgal cover exceeding 67%, frequently obscuring portions of the substrate and the underlying fauna. The other northerly station, FH, had macroalgal cover exceeding 50% at three of its sites. All HI sites had macroalgal cover below 46%, and FH‐s and HI‐s had values below 30%. Across all sites, the macroalgal community was dominated by rhodophyta (notably *Iridaea cordata* , *Trematocarpus antarcticus*, and *Plocamium hookeri*), while phaeophyceae such as *Desmarestia antarctica* and *Himantothallus grandifolius* were present in smaller proportions (see abundance data).

### 3.4 NMDS

**FIGURE 2:**
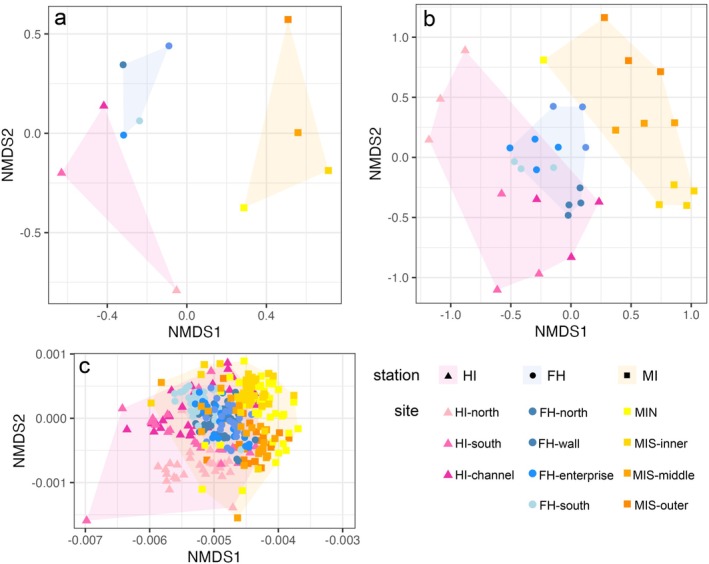
NMDS plots of community data at 3 different scales: (a) unit = site, (b) unit = transect, and (c) unit = image. Stress values of each plot are (a) 0.12, (b) 0.21, and (c) 0.05.

Figure 2 shows the ordination of the sites, transects and images in two‐dimensional NMDS plots. The site‐scale NMDS (Figure 2a) shows a distinction between stations and no apparent overlap. An ANOSIM analysis at site‐scale revealed significant differences between all stations; HI and MI (*R* = 0.444, *p* = 0.029), HI and FH (*R* = 0.519, *p* = 0.029), and MI and FH (*R* = 0.469, *p* = 0.029). The transect‐scale NMDS (Figure 2b) has a less distinct separation of the stations, with an overlap between HI and FH; however, all stations were significantly different with the ANOSIM results giving *R* values of between 0.545 and 0.597 and *p* values of 0.001. In the image‐scale NMDS (Figure 2c), the three stations overlap heavily, with no clear distinction between them and the ANOSIM results showing low R statistics.

### 3.5 Environmental Drivers

**FIGURE 3:**
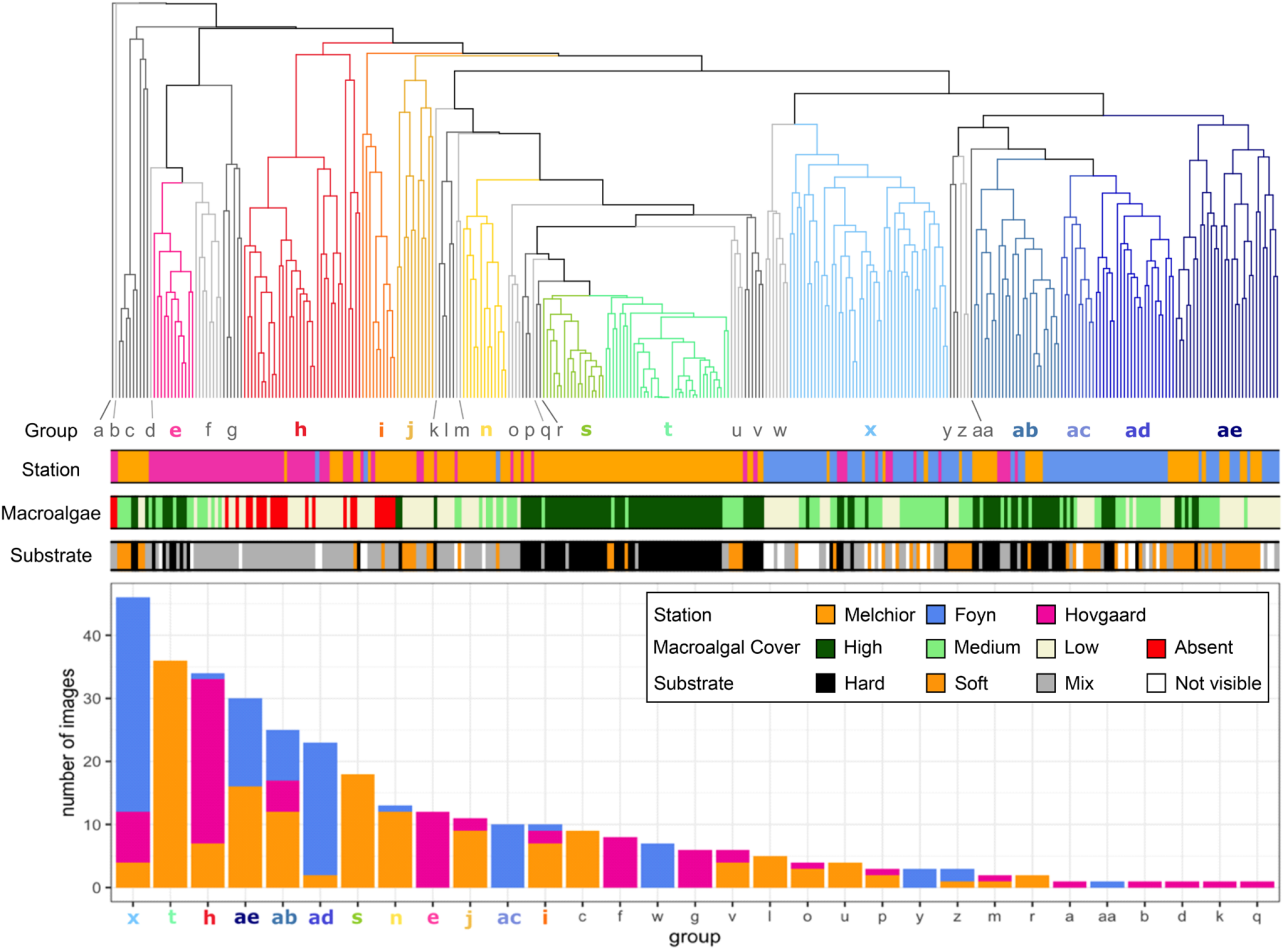
(top) SIMPROF dendrogram showing significant groupings (*p* < 0.05) of benthic images based on community composition, using Bray–Curtis similarity. Groups are colour‐coded, with coloured branches indicating different clusters, and main groups (+10 images) are highlighted. Horizontal bars below the dendrogram indicate sampling station (Melchior Islands, Foyn Harbour, Hovgaard Islands), macroalgal cover (high, medium, low, and absent), and substrate type (hard, soft, mixed, and not visible) of each image. (bottom) Histogram showing the number of images per SIMPROF group, colour‐coded by station.

We tested the relationship between community composition and substrate type using ANOSIM, which yielded a low *R* statistic (*R* = 0.126, *p* = 0.001). Similarly, we found that ANOSIM results based on macroalgae cover also yielded a low *R* statistic (*R* = 0.23, *p* = 0.001). Suggesting that these two factors had little influence at the sub‐meter scale of individual images. This lack of influence is further supported by Figure 3, where the distribution of substrate type and macroalgal cover show that no microhabitats have a single substrate type or macroalgal cover category. Where the substrate type was visible, the macroalgal cover showed a range of categories in each of the substrate classes, meaning that substrate type does not directly correlate with macroalgal cover (Figure 3).

### 3.6 Hierarchical Clustering and SIMPROF Analysis

From our data, 31 significant community composition clusters or microhabitat classes were identified using a SIMPROF analysis to a 5% significance level (Figure 3). The different colours indicate the significant clusters, and they were labelled alphabetically from a to ae. The number of images per microhabitat class varies between 1 and 46 (Figure 3). The 12 most common microhabitat classes (***e***, ***h***, ***i***, ***j***, ***n***, ***s***, ***t***, ***x***, ***ab***, ***ac***, ***ad***, and ***ae***) made up of 10 or more images were further examined through SIMPER analysis and further comparisons, see Table S1 and SIMPER results.

The clusters and histogram (Figure 3) show us that some of these microhabitats are common and widespread, occurring frequently in all three stations (e.g., ***x*** and ***ab***). Other microhabitats are common in one or two stations whilst being rare in the others (e.g., ***h***, ***ad***, ***n***, ***j***, and ***i***). One common microhabitat, ***ae***, is found abundantly in both MI and FH but, along with ***n***, ***s***, ***t***, ***ac***, and ***ad***, is absent from HI. Microhabitat classes ***e***, ***j***, ***s***, and ***t*** are absent from FH, and only two classes, ***e*** and ***ac***, are absent from MI. Four of the 12 most abundant microhabitat classes appear to be restricted to single stations within our study. Microhabitats ***s*** and ***t*** are found only at MI, ***e*** found at HI, and ***ac*** known only from FH.

### 3.7 Main Contributing Taxa to Clusters

**FIGURE 4:**
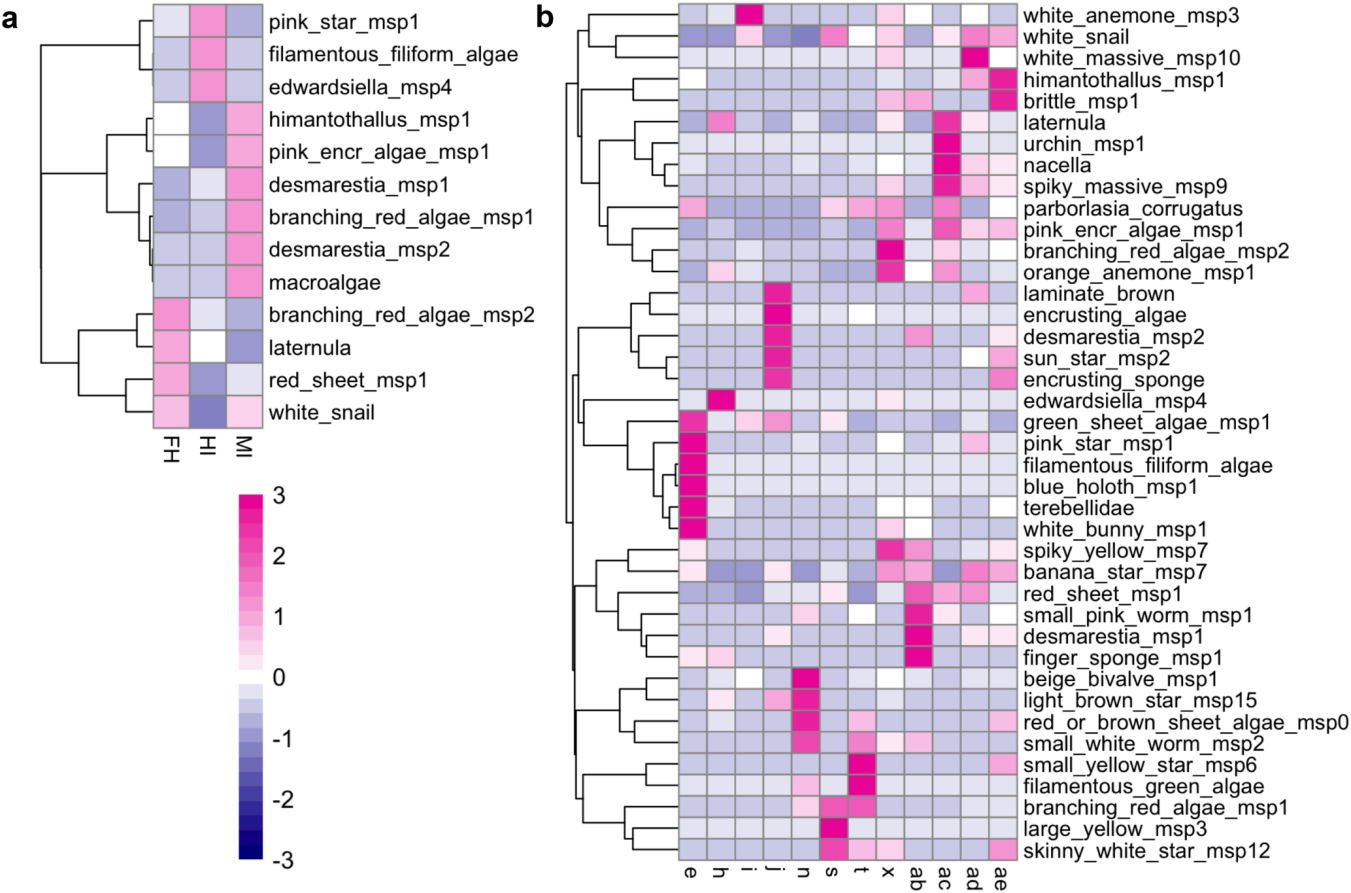
Heatmaps of top contributing taxa to the SIMPER analysis: (a) between stations, (b) between main SIMPROF groups (microhabitats). Colour of the cells represent the standardised abundance of morphotaxa in the group with relative higher abundance in pink and relative lower abundance in blue. The branches on the left side of each heatmap represent how the top defining morphotaxa cluster together in terms of distribution within the stations and microhabitats.

The heatmaps in Figure 4 illustrate how the most influential morphotaxa contribute to the uniqueness of each station and each (main) image group from the SIMPER analyses, with their higher abundance (in pink) or their lower abundance (in blue), see Table S1 and SIMPER results. The branches on the left side of each heatmap represent how the top defining morphotaxa cluster together in terms of distribution within the stations and microhabitats.

From examining the results of the SIMPER (Data S1) and heatmap (Figure 4a) analyses, Melchior Islands' benthic communities differentiate themselves from the other two stations by their higher abundance of *H. grandifolius* (himantothallus_msp1), coralline algae (pink_encr_algae_msp1), *Desmarestia antarctica* (desmarestia_msp1), *Desmarestia anceps* (desmarestia_msp2), *P. hookeri* (branching_red_algae_msp1), and other macroalgae and a low abundance of *I. cordata* (red_sheet_msp1). Hovgaard Islands has less of these algae, but its top contributing taxa are *Edwardsiella andrillae* (edwardsiella_msp4), *Odontaster validus* (pink_star_msp1), and diatom mats (filamentous_filiform_algae) and the absence of *I. cordata* (red_sheet_msp1) (Figure 4a). The absence of *Margarella antarctica* (white_snail) is also notable. Foyn Harbour's communities are characterised by a high abundance in *I. cordata* , *T. antarcticus* (branching_red_algae_msp2), *Laternula elliptica* and *M. antarctica* (Figure 4a). The within station similarity levels were relatively low, between 17% and 34%, and the between station dissimilarity was between 81% and 91%.

Microhabitats that are well separated in the dendrogram (Figure 3, groups ***e*** to ***n***) do not share many influential morphotaxa (SIMPER results in Data S1 and Figure 4b), whereas hierarchically close microhabitats in the dendrogram, such as ***s*** and ***t***, or ***x***, ***ab***, ***ac***, ***ad*** and ***ae***, do share some important defining morphotaxa but in different proportions. A table with descriptions of each microhabitat with top taxa and key images can be found in Table S1 and SIMPER results. The within microhabitat similarity levels were between 28% (***j***) and 83% (***s***), reflecting their position within the dendrogram (Figure 3). Two thirds of the most common microhabitats have within group similarities of over 50%. Dissimilarity levels between stations varied from 25% (microhabitats ***s*** and ***t***) to 97.5% (***t*** and ***e***).

## 4 Discussion

Our results show that the three stations are very different in terms of community compositions and patchiness, although they are not that far apart geographically (Figure 1) and are similar in depth and substrate (Tables 1 and 2). From this coarse spatial scale, it is evident that the three stations are compositionally distinct in their flora and fauna. Yet, a finer‐scale analysis reveals a more nuanced picture of community similarity and variation across scales. Multivariate analyses highlight a strong influence of spatial resolution on community differentiation. When using site as the sampling unit there is a clear separation among the three stations. However, this distinction diminishes with finer resolution. At the finest scale, where image is the sampling unit, the separation between stations becomes indistinct and statistically insignificant (Figure 2). These findings suggest that, in the Northern WAP, averaging at the site—or even transect—level may obscure underlying heterogeneity and similarity between smaller spatial units. These results support the view that ecological patterns are scale‐dependent (Kraan et al. 2015) and align with previous findings that emphasise the importance of multi‐scale approaches or nested designs (Smale 2008; Kraan et al. 2015; Zelnik et al. 2024).

### 4.1 Microhabitats and Patchiness

**FIGURE 5:**
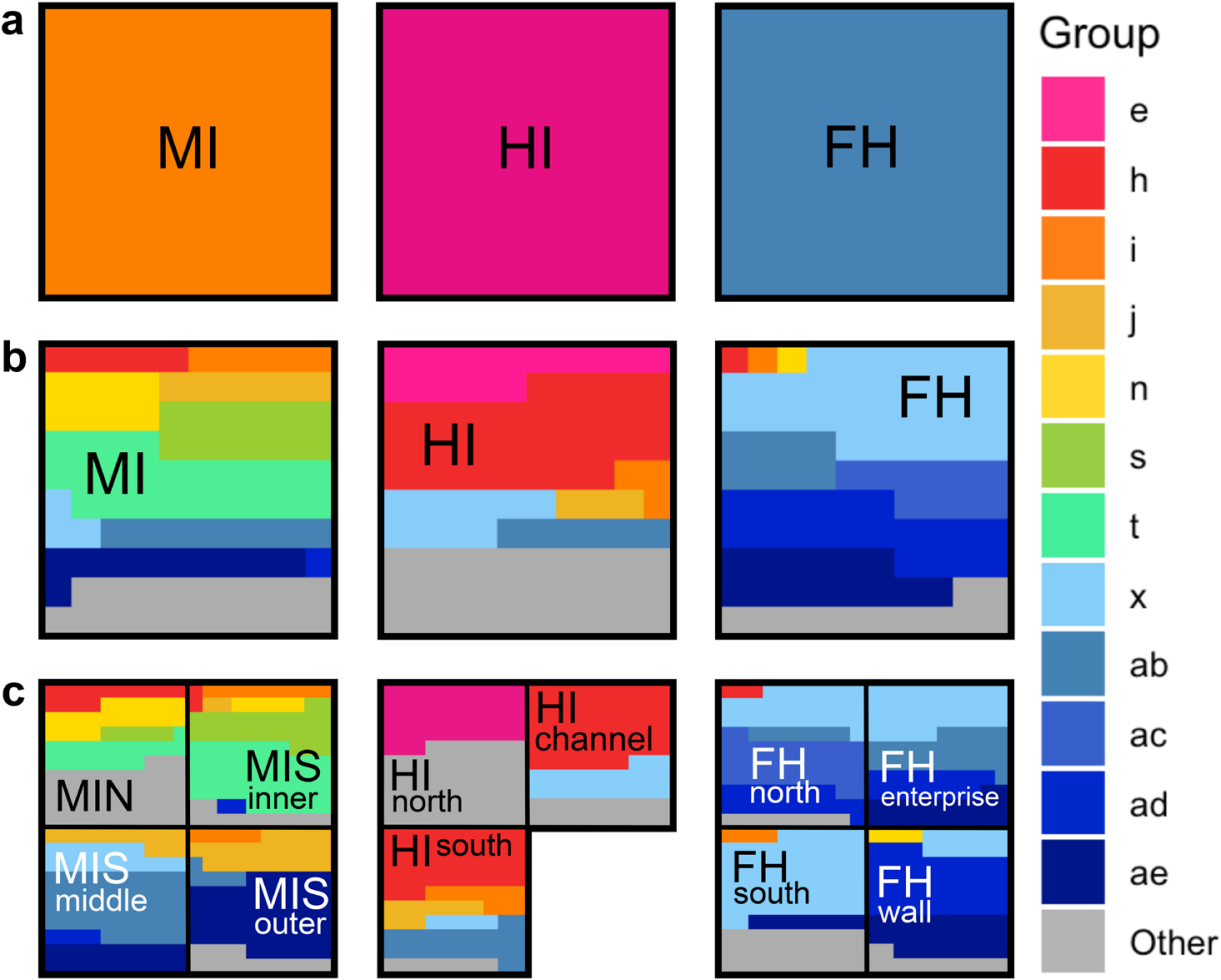
Schematic representation of the effect of spatial scale on benthic community interpretation across our 3 stations. Each larger square represents the total number of images analysed at station (a, b) and site (c) level. Station‐level average community composition (a) is shown as solid colours, representing the distinct separation at this spatial scale. The distribution of the different microhabitat types is given as a percentage of the area of each square (b, c) by colour.

Our analyses identified 31 significant microhabitat classes across all stations, grouped by their similar benthic communities (Figure 4b). Upon further investigation, these microhabitats are not solely driven by geographic separation (Figure 3). The schematic representation in Figure 5 shows us that some of these microhabitats are widespread, occurring frequently in the three stations (e.g., group ***ab***), others are more localised (group ***e*** only found in site HI‐n). Our results also quantify a substantial patchiness between and within both stations and sites (Figure 5). The different proportions of microhabitats within stations and sites imply that the types, abundance and combination of microhabitats are driving the observed differences between localities on both local and larger geographic scales. Each station and site can be considered a mosaic of microhabitats or “ecological units” occurring in different proportions (Figure 5).

The presence and combination of microhabitats within a station or site have a profound influence on their biodiversity metrics and evenness of taxon distribution (Table S2 and Figure S1). Many images from Foyn Harbour, which has the lowest number of microhabitats, are qualified as microhabitat ***x***, which hosts the most biodiverse community and a high 3‐dimensional structural complexity with dense red branching macroalgae and large branching sponges (Figure 5). The fact that all sites in Foyn Harbour include this microhabitat makes the station less heterogeneous but does provide more connectivity for this microhabitat's fauna. In contrast, Melchior Islands has a high diversity of microhabitats (19), including microhabitats that are dominated by single macroalgal species with high percentage cover and lacking in habitat forming animals (***s**, **t*** and ***n***). This high algal cover could exclude sessile invertebrates (Clark et al. 2013), an important and usually dominant component of Antarctic benthos, and lead to an underestimation of abundance and richness of mobile taxa due to the limitations of underwater imagery (that we don't see under the macroalgal canopy). In our study area, having a higher number of microhabitats doesn't necessarily equate to higher diversity (Table S2). Indeed, other factors are important too, such as habitat complexity, connectivity and faunal similarity between microhabitats (Table S1).

While these patterns at the microhabitat level are informative, they also raise important questions about ecological dynamics operating across multiple scales. The finer scale patterns that we highlighted influence biodiversity patterns at wider scales, while wider scale processes can influence small‐scale variability (Teng et al. 2020; Zelnik et al. 2024; Johnston 2024). Another aspect emerging from the SIMPROF clustering is that similar communities can occur across physically distant areas. Much like island systems, microhabitat features such as dropstones have been shown to adhere to the principles of island biogeography (Post et al. 2017; Ziegler et al. 2017). This adherence supports the idea that microhabitat connectivity may be more ecologically relevant than geographical distance alone and likely affects biodiversity and ecosystem resilience (Tielens et al. 2019; Fourcade et al. 2021). This connectivity is important to consider when modelling species distributions or responses of organisms under changing environmental conditions (Keeley et al. 2018; Solà et al. 2024). The temporal dimension is also particularly relevant, as microhabitats may be short lived, shaped by rapid turnover processes such as algal growth cycles or physical disturbances (Momo 2020; Quartino 2020; Amsler et al. 2023). Our results align with several emerging studies, from benthic ecosystems to terrestrial landscapes, that emphasise the importance of cross‐scale interactions and habitat heterogeneity in shaping biodiversity patterns (Thomsen et al. 2022; Zelnik et al. 2024; Barton et al. 2024; Johnston 2024).

### 4.2 Sea Ice and Macroalgal Cover

In the Western Antarctic Peninsula, a region undergoing rapid environmental changes, disruptions in sea‐ice dynamics are known to have increased in the past years (Siegert et al. 2019; Eayrs et al. 2021) and have been proven to affect benthic life (Smale 2008; Pasotti et al. 2015; Amsler et al. 2023). Ice‐related factors, such as sea‐ice concentration and iceberg scouring, were considered in our interpretation of the drivers behind our observations. The North–South sea‐ice gradient in the WAP and associated differences in light availability throughout the year (Amsler et al. 2023) could in part explain why the Hovgaard Islands, which are located slightly more to the south (Figure 1), have a significantly lower macroalgal cover than the other two stations. However, field observations nor available sea‐ice data could explain the meter‐scale variations we observed. Moreover, iceberg scouring was not directly observed or quantified on our transects during our study.

### 4.3 Substrate Type

Another factor we hypothesised was likely influencing the observed patterns is substrate type. Areas with hard substrate such as rocky platforms or dropstones, are generally associated with higher biodiversity (Post et al. 2017; Ziegler et al. 2017; Almond et al. 2021). Given its fundamental role in shaping benthic communities, it is reasonable to expect that differences in substrate composition contribute significantly to the microhabitat variation we documented. While substrate likely plays a role, it does not fully account for the clustering of our microhabitats, as some share substrate types yet differ in benthic composition (***s*** and ***t***), and others (e.g., groups ***c***, ***e***, ***x***, ***ab***, ***ac***, ***ad***, ***ae***) span multiple substrate types (Figure 3). This was further supported by the ANOSIM test, which yielded a low *R* statistic and failed to explain community structure.

### 4.4 Habitat Forming Taxa

The SIMPER analysis (Figure 4b) suggests that macroalgae play a significant role in microhabitat differentiation. A primary hypothesis was that total macroalgal cover would be an important factor in shaping these shallow benthic communities (Figure 3) however our findings (Figure 4b) and the ANOSIM and SIMPER results align more with the assumption that faunal communities are structured by the dominant algae morphotypes that are locally present (Amsler et al. 2015). Some of our microhabitats (e.g., groups ***x***, ***ab***, ***ae***) are associated with complex algal structures (dense forests of *T. antarcticus* , *I. cordata* , or *D. antarctica* ) and the high diversity and abundance of fauna observed in these microhabitats may be linked to the 3D complexity, refugia and varied food source these algae provide (Duarte et al. 2020). Habitat complexity in general is known to increase diversity (Rodil et al. 2021; Thomsen et al. 2022; Leite Jardim et al. 2025). The high abundance of the sponge *Dendrilla antarctica* (spiky_yellow_msp7) in group ***x*** (Figure 4b), in addition to the complex algal beds, is likely behind this group being the most biodiverse type of microhabitat we found. It is important to also consider all habitat forming taxa such as sponges, as they are widely known as foundation species in the Antarctic, both in coastal and deep ecosystems (Downey et al. 2012; Gutt et al. 2017; Mitchell et al. 2020).

### 4.5 Climate Change

In the context of a warming climate, the relevance of these findings becomes even more apparent. The West Antarctic Peninsula (WAP) is one of the most rapidly changing regions on Earth (Turner et al. 2014), and shallow marine ecosystems there have witnessed the most observed changes recently and are predicted to continue to change over coming decades (Griffiths et al. 2024). Increasing temperatures and winds are causing disruptions in sea ice dynamics; sea ice loss and the associated shifts in light availability have been shown to have an extensive impact on benthic communities (Cummings et al. 2006; Clark et al. 2013, 2017; Gutt et al. 2019). Macroalgal cover has been shown to increase with less sea ice cover (Clark et al. 2013; Amsler et al. 2023), suggesting that future changes in ice regimes could significantly alter the presence and identity of dominant algal species. These changes could, in turn, reshape the associated faunal communities (Clark et al. 2013). The northern parts of the WAP—such as the sites included in this study—already experience less consistent and shorter sea ice cover compared to more southern regions (Stammerjohn et al. 2008, 2012). As sea ice continues to decline, these northern locations may offer a preview of conditions likely to develop further south of the Peninsula (Clark et al. 2013; Amsler et al. 2023). The benthic community patterns we observe in this study could foreshadow broader shifts in community structure and habitat compositions as similar conditions expand southward.

On the other hand, habitat diversity and microhabitat mosaics could be crucial for providing connectivity, redundancy and thus resilience under rapid environmental changes (O'Leary et al. 2017; Pearson et al. 2021). Because our study sites exhibit no direct human disturbance, they provide an ideal setting to monitor indirect effects of global environmental change. Studying microhabitat structure in such undisturbed systems offers a sensitive lens to identify subtle ecological shifts that may precede larger‐scale changes.

## 5 Conclusion

Our nested sampling design provides a rare multi‐scale perspective, effectively capturing fine‐scale biodiversity patterns while allowing comparisons across more extensive spatial scales. Our study shows that the shallow benthic communities of the West Antarctic Peninsula cannot be considered uniform habitats but rather mosaics of microhabitats, each characterised by distinct macroalgal communities and associated faunal assemblages. This patchwork effect demonstrates the importance of scale, with connectedness and similarity within and between locations being different at different resolutions. Results show that types, diversity and combinations of habitat forming species, including macroalgae and sponges, were more important than macroalgal cover and substrate type in determining microhabitat assemblages. Sea ice dynamics are a likely driver of the presence and composition of algal species and associated faunal communities, but sea ice data is currently unavailable at site and microhabitat relevant scales.

Microhabitats were found to be distinct and could be location specific or shared across multiple locations. The number and types of microhabitats present in each region drive the overall biodiversity and biogeographic signature of each location. Monitoring changes at the microhabitat scale could serve as an early warning system for broader ecological shifts undetectable at the station scale, particularly in regions undergoing rapid environmental change.

## Author Contributions

**Lea Katz:** conceptualization (equal), data curation (lead), formal analysis (lead), investigation (lead), methodology (lead), visualization (lead), writing – original draft (lead). **Emily Mitchell:** conceptualization (equal), project administration (equal), supervision (equal), validation (equal), writing – review and editing (equal). **Bruno Danis:** conceptualization (equal), funding acquisition (lead). **Huw Griffiths:** conceptualization (equal), formal analysis (supporting), investigation (supporting), methodology (supporting), project administration (equal), supervision (equal), validation (equal), visualization (supporting), writing – review and editing (equal).

## Funding

Lea Katz and Bruno Danis are funded by the TANGO project funded by the Belgian Federal Science Policy Office (B2/212/P1/TANGO). Emily Mitchell is funded as part of the “Ediacaran constraints on early animal evolution fellowship” (NE/S014756/1) from Natural Environment Research Council (NERC). Huw Griffiths is funded by NERC through the “BIOPOLE” Project (NE/W004933/1) and the “The past, present and future of unique cold‐water benthic (sea floor) ecosystems in the Southern Ocean” fellowship (MR/W01002X/1).

## Conflicts of Interest

The authors declare no conflicts of interest.

## Supporting information


**Table S1:** Description and characteristics of most abundant SIMPROF groups.
**Table S2:** Summary table of station biodiversity indexes.
**Figure S1:** Boxplots of biodiversity metrics of studied sites. (a) Shannon–Wiener diversity index. (b) Pielou's evenness index. (c) Number of different morphotaxa. (d) Number of different microhabitats.
SIMPER Analysis results on microhabitat groupings

## Data Availability

Raw abundance data is available at: https://doi.org/10.5061/dryad.stqjq2cj3 and https://doi.org/10.5281/zenodo.16736897.
