# Supplementary material for: Microhabitat Patchiness Structures Benthic Biodiversity in the Western Antarctic Peninsula
Source: Ecol Evol. 2026 Apr 1;16(4):e73392. doi: 10.1002/ece3.73392 (PMC13045378; doi:10.1002/ece3.73392)
Supplement: Supplementary file 1 — Table S1: Description and characteristics of most abundant SIMPROF groups. Table S2: Summary table of station biodiversity indexes. Figure S1: Boxplots of biodiversity metrics of studied sites. (a) Shannon–Wiener diversity index. (b) Pielou's evenness index. (c) Number of different morphotaxa. (d) Number of different microhabitats. SIMPER Analysis results on microhabitat groupings [file ECE3-16-e73392-s001.docx]

**Supporting Information for:**

**Microhabitat patchiness structures benthic biodiversity in the Western Antarctic Peninsula**

Lea Katz, Emily Mitchell, Bruno Danis, Huw Griffiths

*Supplementary Table 1 - Description and characteristics of most abundant SIMPROF groups*

| Group | Found in | Description  (For most common morphotaxa, most probable identification is put between brackets) | Total n of taxa | Mean n of taxa per image | Key taxa (SIMPER) | Key image |
| --- | --- | --- | --- | --- | --- | --- |
| **e** | MI: 0  HI: 22  FH: 0 | Dominated by rocky substrate and large amounts of diatom mats. Faunal community composed mainly by *Odontaster validus* (pink_star_mps1), *Heterocucumis steineni* (blue_holoth_msp1), terebellid worms and *Doris kerguelenensis* (white_bunny_msp1). Notable lack of *Margarella antarctica* (white_snail) and *Laternula elliptica* compared to the other groups. Groups ***d***, ***f*** and ***g*** are very similar to ***e*** but have some rarer species within their images. | 23 | 5.75 | Macroalgae:   filamentous_filiform_algae  Fauna:  pink_star_msp1 blue_holoth_msp1 terebellidae white_bunny_msp1 | 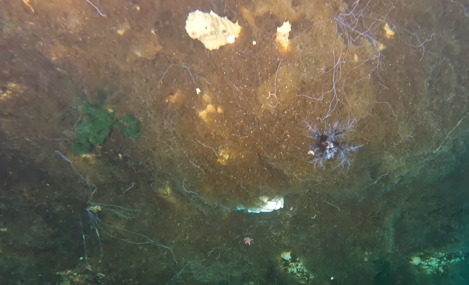TANGO2_ROV13_2_027.png (HI) |
| **h** | MI: 6  HI: 47  FH: 1 | Typical soft bottom habitat. High abundance in *L. elliptica* and *Edwardsiella andrillae,* and just like group ***e***, a distinct lack of *M. antarctica.* | 20 | 3.23 | Very little macroalgae, no dominant one Fauna: edwardsiella_msp4 laternula | 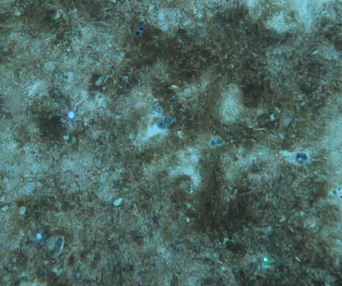TANGO2_ROV14_7_16.png (HI) |
| **i** | MI: 6  HI: 4  FH: 1 | Also soft bottom, but faunal community of group ***i*** is very different from group ***h***. The most abundant morphotaxa is white_anemone_msp3 and *M. antarctica* is also more frequently present, but the total number of taxa in this group is very low (9). | 9 | 2.2 | white_anemone_msp3 | 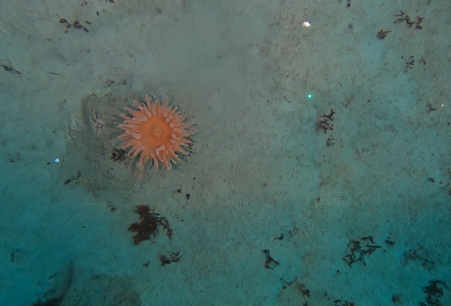TANGO2_ROV19_1_045.png (FH) |
| **j** | MI: 7  HI: 4  FH: 0 | Many different macroalgae and starfish morphotaxa. | 18 | 3.36 | Macroalgae: laminate_brown encrusting_algae desmarestia_msp2Fauna:  sun_star_msp2 encrusting_sponge | 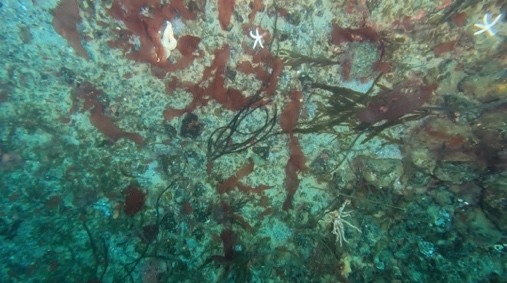TANGO2_ROV10_3_004.png (MI) |
| **n** | MI: 10  HI: 0  FH: 1 | Images from group ***n*** all have some amount of *Plocamium hookeri* (branching_red_algae_msp1). The faunal community is quite cryptic, with some light_brown_star_msp15 and beige_bivalve_msp1, blending in with the sandy bottom seascape. | 15 | 3.61 | Macroalgae: red_or_brown_sheet_algae_msp0 filamentous_green_algae Fauna:  beige_bivalve_msp1 light_brown_star_msp15 small_white_worm_msp2 | 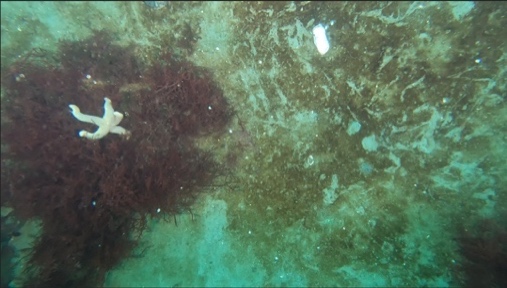TANGO2_ROV9_1_028.png (MI) |
| **s** & **t** | (s)  MI: 15  HI: 0  FH: 0  (t)  MI: 29  HI: 0  FH: 0 | Groups ***s*** and ***t*** are very similar in their macroalgae community composition. The dominant algae morphotaxa is *P. hookeri*, in much higher density then group ***n***. The difference between the two groups is the faunal composition living within the dense bushes. | 12 (s) & 22 (t) | 3.44 (s) & 2.63 (t) | Macroalgae:  branching_red_algae_msp1 Fauna:  large_yellow_msp3 skinny_white_star_msp12 white_snail small_yellow_star_mps6 skinny_white_star | 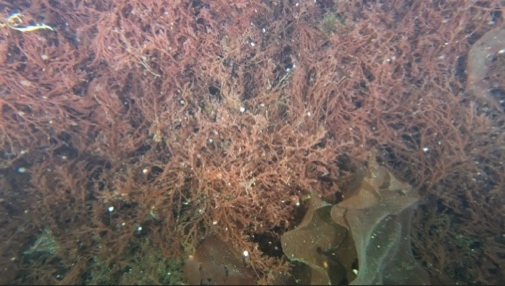TANGO2_ROV6_1_028.png (MI) |
| **x** | MI: 3  HI: 15  FH: 38 | Very diverse macroalgae bushes with sponges (*Dendrilla antarctica*) and anemones. | 44 | 6.69 | Macroalgae: branching_red_algae msp2 Fauna: spiky_yellow_msp7 orange anemone_msp1 | 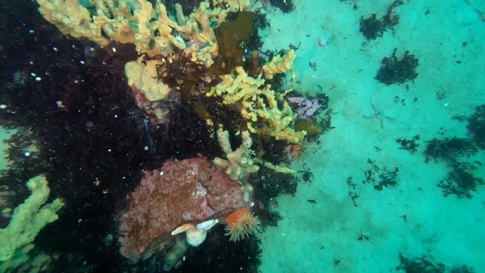 TANGO2_ROV14_2_046.png (HI) |
| **ab** | MI: 10  HI:9  FH: 9 | Very diverse macroalgae forest, although different from group ***j***, with a high abundance of *Iridaea cordata* (red_sheet_msp1) and *Desmarestia antarctica* (desmarestia_msp1). A common feature with group ***x*** is the high abundance of *Dendrilla antarctica*. | 26 | 3.8 | Macroalgae: desmarestia_msp1 red_sheet_msp1Fauna: finger_sponge_msp1 small_pink_worm_msp1 | 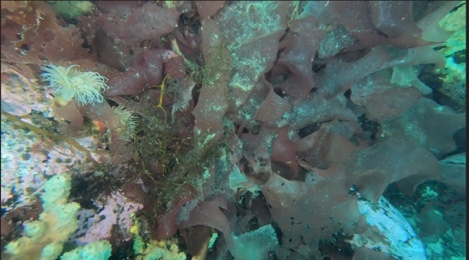TANGO2_ROV20_3_013.png (FH) |
| **ac** | MI: 0  HI: 0  FH: 11 | Groups ***ab*** and ***ac*** cluster together due to their high abundance of *I. cordata*, but group ***ac***’s faunal community is very distinct from ***ab***. The most influential morphotaxa distinguishing this group from the others are *Sterechinus neumayeri* (urchin_msp1) and *Nacella concinna.* Main substrate type is rock or other consolidated types, covered by pink_encr_algae_msp1 (probably *Leptophytum coulmanicum*). | 18 | 8 | Macroalgae: red_sheet_msp1 pink_encr_algae Fauna:  laternula urchin_msp1 nacella spiky_massive_msp9 parborlasia_corrugatus | 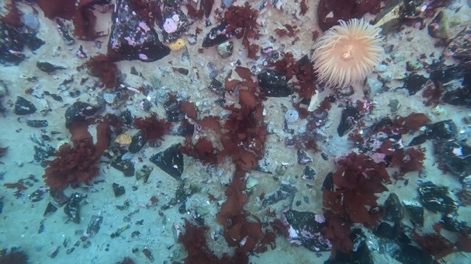TANGO2_ROV16_2_026.png (FH) |
| **ad** | MI : 2  HI: 0  FH: 23 | Similarly to group ***ab*** and ***ac***, the abundant *I. cordata* is one of the defining morphotaxa of group ***ad***. Some fauna in common with group ***ac***, although less dominant (this is the case for *N. concinna*, spiky_massive_msp9, pink_encr_algae_msp1 and *L. elliptica*). The most contributing morphotaxa of this group to the differences with the other groups is the high abundance of the sponge morphotype white_massive_msp10. | 30 | 6.69 | Macroalgae: red_sheet_msp1 Fauna: white_massive_msp10 white_snail | 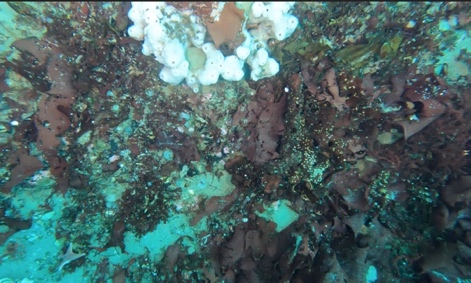TANGO2_ROV21_1_065.png (FH) |
| **ae** | MI: 13  HI: 0  FH: 16 | Images from this group distinguish themselves from the others by their high cover of *Himantothallus grandifolius* and high abundance of brittle stars. | 41 | 7.06 | Macroalgae: himatothallus_msp1 Fauna:  brittle_msp1 encrusting_sponge Other stars | 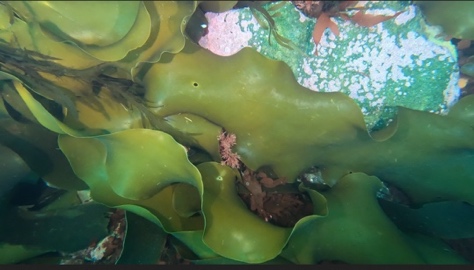TANGO2_ROV10_4_013.png (MI) |

*Supplementary Table 2 – Summary table of station biodiversity indexes*

| Station | Number of  morphotaxa | Shannon-Wiener  diversity | Pielou’s evennes | Number of  microhabitats |
| --- | --- | --- | --- | --- |
| **HI** | 45 | 2.326 | 0.611 | 17 |
| **MI** | 53 | 1.611 | 0.405 | 19 |
| **FH** | 50 | 2.117 | 0.541 | 12 |


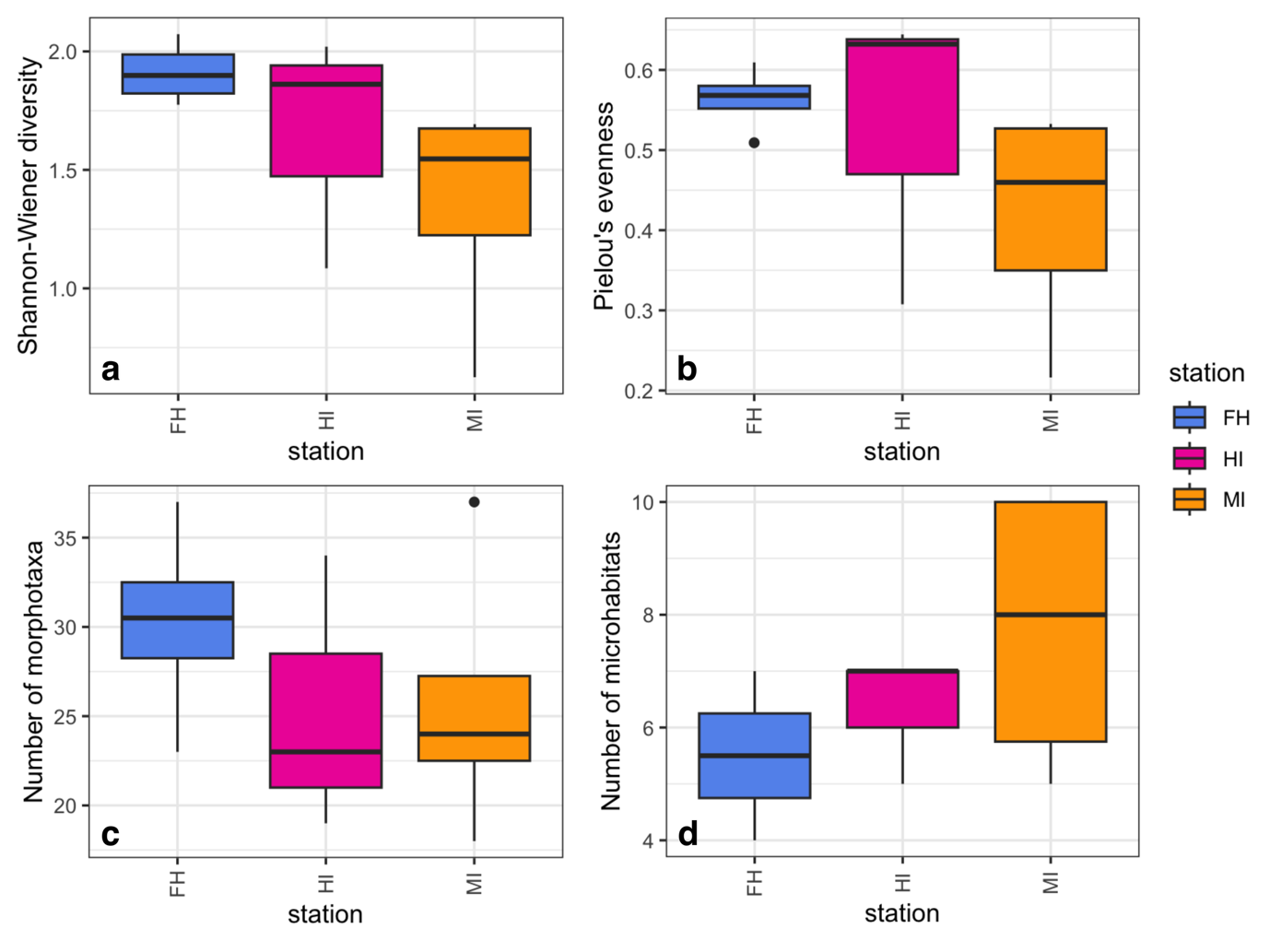


*Supplementary Figure 1 - Boxplots of biodiversity metrics of studied sites. (a) Shannon-Wiener diversity index. (b) Pielou’s evenness index. (c) Number of different morphotaxa. (d) Number of different microhabitats*

**SIMPER Analysis results on microhabitat groupings:**

Microhabitat

Similarity Percentages - Morphotaxa contributions

One-Way Analysis

*Parameters*

Resemblance: S17 Bray-Curtis similarity

Cut off for low contributions: 70.00%

**Between Stations:**

| *Group MI* |  |  |  |  |  |
| --- | --- | --- | --- | --- | --- |
| Average similarity: 29.66 |  |  |  |  |  |
|  |  |  |  |  |  |
| Species | Av.Abund | Av.Sim | Sim/SD | Contrib% | Cum.% |
| branching_red_algae_msp1 | 4.87 | 18.62 | 0.72 | 62.78 | 62.78 |
| red_sheet_msp1 | 2.01 | 6.52 | 0.73 | 21.97 | 84.75 |
|  |  |  |  |  |  |
| *Group HI* |  |  |  |  |  |
| Average similarity: 17.21 |  |  |  |  |  |
|  |  |  |  |  |  |
| Species | Av.Abund | Av.Sim | Sim/SD | Contrib% | Cum.% |
| edwardsiella_msp4 | 0.97 | 3.71 | 0.41 | 21.58 | 21.58 |
| laternula | 1.06 | 3.63 | 0.35 | 21.1 | 42.68 |
| filamentous_filiform_algae | 1.99 | 3.26 | 0.29 | 18.93 | 61.61 |
| red_sheet_msp1 | 1.28 | 2.24 | 0.37 | 13.03 | 74.63 |
|  |  |  |  |  |  |
| *Group FH* |  |  |  |  |  |
| Average similarity: 34.07 |  |  |  |  |  |
|  |  |  |  |  |  |
| Species | Av.Abund | Av.Sim | Sim/SD | Contrib% | Cum.% |
| red_sheet_msp1 | 3.94 | 12.07 | 0.94 | 35.42 | 35.42 |
| branching_red_algae_msp2 | 2.69 | 6.64 | 0.65 | 19.48 | 54.91 |
| white_snail | 1.99 | 6.33 | 0.97 | 18.58 | 73.49 |

Dissimilarity matrix:

|  | MI | HI |
| --- | --- | --- |
| MI |  |  |
| HI | 90.8 |  |
| FH | 81.2 | 85.7 |

**Between main SIMPROF groups (microhabitats):**

| *Group ab* |  |  |  |  |  |
| --- | --- | --- | --- | --- | --- |
| Average similarity: 56.13 |  |  |  |  |  |
|  |  |  |  |  |  |
| Species | Av.Abund | Av.Sim | Sim/SD | Contrib% | Cum.% |
| red_sheet_msp1 | 8.79 | 51.57 | 3.89 | 91.89 | 91.89 |
|  |  |  |  |  |  |
| *Group ae* |  |  |  |  |  |
| Average similarity: 38.06 |  |  |  |  |  |
|  |  |  |  |  |  |
| Species | Av.Abund | Av.Sim | Sim/SD | Contrib% | Cum.% |
| red_sheet_msp1 | 2.34 | 11.9 | 1.83 | 31.28 | 31.28 |
| white_snail | 2.36 | 10.38 | 1.38 | 27.28 | 58.56 |
| himantothallus_msp1 | 3.45 | 8.97 | 0.65 | 23.58 | 82.14 |
|  |  |  |  |  |  |
| *Group j* |  |  |  |  |  |
| Average similarity: 27.52 |  |  |  |  |  |
|  |  |  |  |  |  |
| Species | Av.Abund | Av.Sim | Sim/SD | Contrib% | Cum.% |
| red_sheet_msp1 | 1.86 | 19.05 | 1.27 | 69.2 | 69.2 |
| desmarestia_msp2 | 2.36 | 7.05 | 0.55 | 25.62 | 94.83 |
|  |  |  |  |  |  |
| *Group i* |  |  |  |  |  |
| Average similarity: 51.36 |  |  |  |  |  |
|  |  |  |  |  |  |
| Species | Av.Abund | Av.Sim | Sim/SD | Contrib% | Cum.% |
| white_snail | 1.73 | 48.77 | 2.75 | 94.96 | 94.96 |
|  |  |  |  |  |  |
| *Group e* |  |  |  |  |  |
| Average similarity: 68.10 |  |  |  |  |  |
|  |  |  |  |  |  |
| Species | Av.Abund | Av.Sim | Sim/SD | Contrib% | Cum.% |
| filamentous_filiform_algae | 8.64 | 49.59 | 6 | 72.82 | 72.82 |
|  |  |  |  |  |  |
| *Group h* |  |  |  |  |  |
| Average similarity: 40.81 |  |  |  |  |  |
|  |  |  |  |  |  |
| Species | Av.Abund | Av.Sim | Sim/SD | Contrib% | Cum.% |
| laternula | 2.73 | 23.76 | 1.26 | 58.23 | 58.23 |
| edwardsiella_msp4 | 1.79 | 13.81 | 0.93 | 33.85 | 92.07 |
|  |  |  |  |  |  |
| *Group x* |  |  |  |  |  |
| Average similarity: 46.14 |  |  |  |  |  |
|  |  |  |  |  |  |
| Species | Av.Abund | Av.Sim | Sim/SD | Contrib% | Cum.% |
| branching_red_algae_msp2 | 5.92 | 28.93 | 2.46 | 62.7 | 62.7 |
| white_snail | 1.85 | 5.09 | 0.85 | 11.03 | 73.73 |
|  |  |  |  |  |  |
| *Group ad* |  |  |  |  |  |
| Average similarity: 54.71 |  |  |  |  |  |
|  |  |  |  |  |  |
| Species | Av.Abund | Av.Sim | Sim/SD | Contrib% | Cum.% |
| red_sheet_msp1 | 6.39 | 31.41 | 3.94 | 57.41 | 57.41 |
| white_snail | 2.88 | 13.22 | 2.69 | 24.16 | 81.57 |
|  |  |  |  |  |  |
| *Group ac* |  |  |  |  |  |
| Average similarity: 60.44 |  |  |  |  |  |
|  |  |  |  |  |  |
| Species | Av.Abund | Av.Sim | Sim/SD | Contrib% | Cum.% |
| red_sheet_msp1 | 6 | 19.94 | 3.14 | 32.99 | 32.99 |
| laternula | 4.01 | 14.14 | 1.57 | 23.4 | 56.39 |
| urchin_msp1 | 2.85 | 11.95 | 3.82 | 19.77 | 76.16 |
|  |  |  |  |  |  |
| *Group n* |  |  |  |  |  |
| Average similarity: 60.58 |  |  |  |  |  |
|  |  |  |  |  |  |
| Species | Av.Abund | Av.Sim | Sim/SD | Contrib% | Cum.% |
| branching_red_algae_msp1 | 4.27 | 40.26 | 3.36 | 66.46 | 66.46 |
| red_sheet_msp1 | 2.13 | 15.76 | 1.37 | 26.02 | 92.49 |
|  |  |  |  |  |  |
| *Group t* |  |  |  |  |  |
| Average similarity: 82.65 |  |  |  |  |  |
|  |  |  |  |  |  |
| Species | Av.Abund | Av.Sim | Sim/SD | Contrib% | Cum.% |
| branching_red_algae_msp1 | 9.71 | 77.26 | 9.84 | 93.48 | 93.48 |
|  |  |  |  |  |  |
| *Group s* |  |  |  |  |  |
| Average similarity: 83.09 |  |  |  |  |  |
|  |  |  |  |  |  |
| Species | Av.Abund | Av.Sim | Sim/SD | Contrib% | Cum.% |
| branching_red_algae_msp1 | 9.74 | 55.68 | 7.97 | 67.01 | 67.01 |
| red_sheet_msp1 | 3.31 | 16.96 | 4.94 | 20.41 | 87.42 |

Dissimilarity matrix:

|  | e | h | i | j | n | s | t | x | ab | ac | ad |
| --- | --- | --- | --- | --- | --- | --- | --- | --- | --- | --- | --- |
| e |  |  |  |  |  |  |  |  |  |  |  |
| h | 97.2 |  |  |  |  |  |  |  |  |  |  |
| i | 96.9 | 91.9 |  |  |  |  |  |  |  |  |  |
| j | 95.4 | 93.4 | 94.4 |  |  |  |  |  |  |  |  |
| n | 94.5 | 88.2 | 95.9 | 81.5 |  |  |  |  |  |  |  |
| s | 94.1 | 93.1 | 85.3 | 84 | 50.8 |  |  |  |  |  |  |
| t | 97.5 | 95.7 | 87.9 | 95.2 | 55.8 | 25.8 |  |  |  |  |  |
| x | 92.4 | 85.6 | 84.3 | 89.7 | 84.6 | 79.5 | 88.3 |  |  |  |  |
| ab | 93 | 93.8 | 95.2 | 78.9 | 78.1 | 74.8 | 92.4 | 82.2 |  |  |  |
| ac | 93.3 | 77.9 | 87 | 84.9 | 80.2 | 75.5 | 91.1 | 68.6 | 66.3 |  |  |
| ad | 89.6 | 87.1 | 79.6 | 81.3 | 73.1 | 62 | 81.8 | 71.1 | 58 | 56.2 |  |
| ae | 92 | 90 | 79.2 | 81.4 | 77.4 | 70.1 | 83 | 74.9 | 78.6 | 71.3 | 62.5 |
